# Traumatic Macroglossia in a Patient With Rett Syndrome

**DOI:** 10.7759/cureus.26172

**Published:** 2022-06-21

**Authors:** Anass Dweik, Yasir Al-Hilli, Yousuf Tawfeeq, Job Varghese, Thien Vo

**Affiliations:** 1 Internal Medicine, Texas Tech University Health Sciences Center, Amarillo, USA; 2 Pulmonology and Critical Care, Texas Tech University Health Sciences Center, Amarillo, USA

**Keywords:** critical care, airway obstruction, rett syndrome, macroglossia, traumatic macroglossia

## Abstract

Traumatic macroglossia is a rare condition characterized by a sudden edematous swelling of the tongue due to trauma that can progress into upper airway obstruction and asphyxia. We are presenting a case of a 20-year-old female with medical history significant for Rett syndrome who developed severe tongue swelling after multiple attempts of intubation secondary to low Glasgow Coma Scale (GCS) from a high dose of benzodiazepines. Traumatic macroglossia in this case was worsened further by uncontrolled bruxism. Multiple approaches were made to control the situation including placement of bite block, use of paralytics, and steroids. Multiple cases were reported about traumatic macroglossia but our case was unique in the sense that our patient did not respond well to conservative medical therapy and surgical approach was not possible as it was set to be done in a tertiary center; however, transferring the patient was not possible with the COVID-19 pandemic as hospitals were on diversion.

## Introduction

Macroglossia is a condition in which the tongue’s size is enlarged beyond the alveolar ridge or teeth. It is differentiated from glossoptosis, a condition defined as having a relative macroglossia, by having a relatively normal-sized oral cavity and a true enlargement of the tongue [1]. It is associated with multiple conditions; some are congenital and others are acquired. Some of the most commonly associated conditions include congenital hypothyroidism, Beckwith-Wiedmann syndrome, primary amyloidosis, Down syndrome, and acromegaly [2]. Traumatic macroglossia is a rarely reported condition that is most significant for its rapidity and life-threatening complications; most importantly is upper airway obstruction and asphyxia. 

## Case presentation

**Figure 1:**
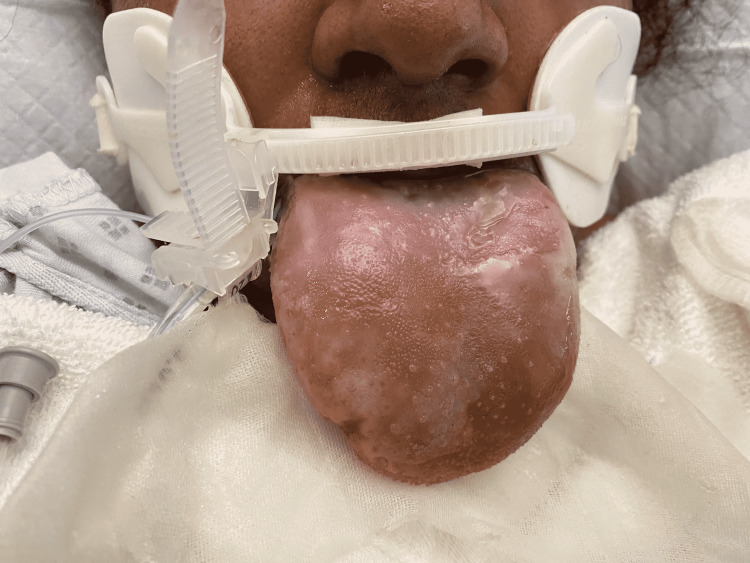
extensive diffuse enlargement of the tongue as seen on anterior view. Necrotic tissue along with relatively pale tongue can also be observed that is likely due to the lack of blood supply caused by the trauma.

**Figure 2:**
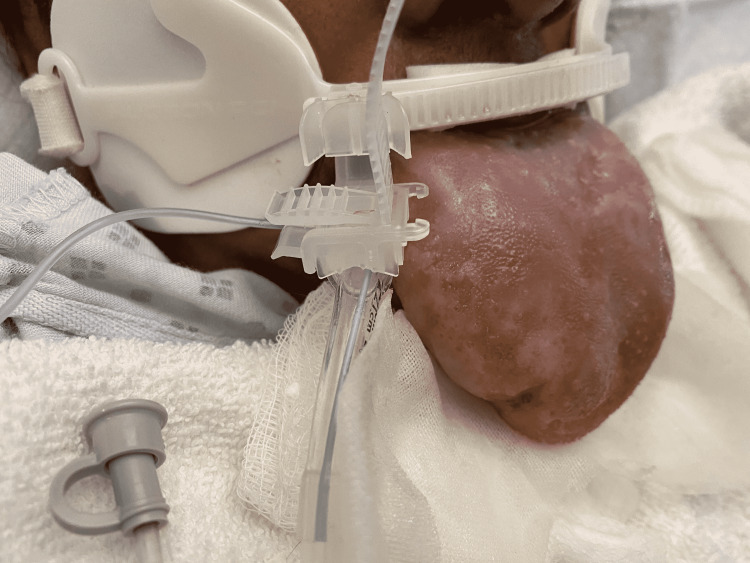
Lateral view of the tongue

A 20-year-old female with medical history significant for Rett syndrome, spastic paraplegia, and seizure disorder was admitted to the hospital following benzodiazepine overdose and complications post intubation. As per the medical records, patient had a dental appointment for which she was given a high dose of benzodiazepine that lead to over-sedation. She required to be intubated as her Glasgow Coma Scale (GCS) was low; however, multiple attempts were made with a direct laryngoscope while awaiting transporting the patient from the dental clinic to the hospital as most hospitals were on diversion due to the COVID-19 pandemic that led to laryngeal edema and swelling of her tongue. She was finally intubated after having a laryngeal mask airway (LMA) for more than 12 hours. Even after a few days, her significantly enlarged tongue raised concerns about difficult extubation. The patient continued clenching of her teeth worsened the tongue trauma (Figures 1, 2) and an ENT consultation was placed for the same who recommended a bite block and dexamethasone. Hence, our patient received the aforementioned recommendations but her tongue swelling continued to be unchanged. After a detailed discussion with the family, a decision was made to start her on paralytic to help reduce the pressure on her tongue, which can help with recovery. She was kept on a paralytic for more than a week and there was no improvement in tongue size. ENT recommended tracheostomy at a tertiary center as the patient’s tongue showed no improvement following all conservative measures and a possible partial glossectomy in efforts of opening the upper airway. Since all the hospitals in the region were on diversion due to the COVID-19 pandemic, she continued to stay in our hospital after multiple attempts for transfer. Later, family approached the medical team to update goals of care to reflect palliative care/hospice given her overall poor prognosis from both Rett syndrome and the current issue of traumatic macroglossia. Patient was switched to DNR/DNI and terminal extubation was done. Unfortunately, she passed away a few hours after being under hospice care.

## Discussion

Macroglossia is defined as an enlargement of the tongue beyond its normal surface area. It is differentiated from glossoptosis by the presence of a true tongue enlargement as compared to a relative size difference that is seen in the latter. Macroglossia is one of the few conditions that can be overseen when it comes to its significance to obstructing the upper airway. Any upper airway obstruction is considered to be an emergency leading to a rapid intervention. In macroglossia, the upper airway obstruction may at times be more of a subtle change happening over hours to days masking the rapid response that is needed to protect the airway, especially in the younger/healthier population where it may take longer for hypoxia/hypoxemia to be observed clinically. Traumatic macroglossia is not a common condition. In fact, there are only a few case reports that discuss this issue and it is not often found in common references as a subtype of macroglossia. In addition, there are also cases of mixed macroglossia that can happen when a previously noted macroglossia progresses significantly turning into an alarmingly obstructive macroglossia secondary to trauma or anaphylaxis. As for the treatment of traumatic macroglossia, there are multiple recommended therapies that have been used previously to help lessen the stress on the tongue and relieve upper airway obstruction. Some of these measures were to massage the tongue as described by Saah et al. [3]. The concept behind that is to help lymphatic and venous drainage by relieving the pressure on the tongue. This approach was not possible in our case as our patient was not able to follow commands even after giving her a sedation vacation. Another approach that was described was to place a tongue bite raiser to help prevent active tongue biting as described by Roberts et al. [4]. This measure was attempted on our patient; however, no improvement was seen even a week following bite raiser placement. Alvi et al. also recommended the utilization of wet dressing in addition to a bite block and hand massage in efforts to buy time to prevent tongue necrosis [5]. Finally, Lamond et al. suggested significant results after steroid injections into the base of the traumatic tongue. In their case, they injected 200mg of intramuscular triamcinolone on the two lateral ends and one was injected in the midline of the tongue which showed significant improvement in the blood flow of the tongue within 48 hours of injection [6]. There are some other reported cases where tracheostomy was performed after a week of conservative therapy as the tongue swelling was not improving [3,7].

## Conclusions

Traumatic macroglossia is a rare yet serious condition that requires rapid intervention to help secure the upper airway and retrieve as much tissue as possible from the tongue. Multiple treatment modalities have been suggested including bite block, tongue massage, wet dressings, intramuscular steroid administration, and finally tracheostomy in severe cases with or without glossectomy.
